# Distribution and risk factors of cleft lip and palate on patients from a sample of Damascus hospitals - A case-control study

**DOI:** 10.1016/j.heliyon.2021.e07957

**Published:** 2021-09-07

**Authors:** Louei Darjazini Nahas, Omar Alzamel, Mammdouh Yassin Dali, Rama Alsawah, Ahmad Hamsho, Rafi Sulman, Mohamad Alzamel, Abdullah Omar

**Affiliations:** a Department of Surgery Division of Otorhinolaryngology, Faculty of Medicine, Syrian Private University, Damascus, Syria; b Faculty of Medicine, Syrian Private University, Damascus, Syria; c Resident at Internal Medicine Department, Damascus University, Damascus, Syria; d Department of Otorhinolaryngology, Damascus, Syria

**Keywords:** Cleft lip, Cleft palate, Down's syndrome, Van der Woude syndrome, Pierre Robin's syndrome, Syria

## Abstract

**Objective:**

This case-control study was conducted to determine the distribution of cleft lip and/or palate, its association with family history, syndromes and serous otitis media (SOM), and its relation with several risk factors.

**Methods:**

The case group comprised of 133 children born with cleft lip and/or palate, and the control was 133 non-cleft children born full-term. Data was collected including age, gender, origin and risk factors for cleft lip and palate from patients’ files, interviewing supervising doctors, and the patient. Data was then filled out into Excel and underwent statistical analysis using the Goodness of Fit Test and Chi-Square to determine the significance of the results.

**Results:**

Cleft lip and/or palate (CL/P) was slightly higher among males (51.9%). Combined cleft lip and palate (CLP) was the most common presentation (42.1%). Cleft lips (CL) were mostly complete cleft (51,5%) incomplete cleft comprised (41.1%), In the sample 35.4% of the cases were bilateral, 32.3% were right unilateral, 28.3% were left unilateral and 4% were median cleft. Cleft palate (CP) was mostly complete (46.6%) there were incomplete clefts (40%), and the remainder were submucosal (13.4%). Isolated CL and combined CLP were higher in males (51.6%, 62.5% respectively). Both isolated CP and Tessier anomaly were more common in females (64.7% and 58.3% respectively). consanguineous marriages accounted for 36.1% of cases. 21.8% of the sample had a first-degree relative and 24.8% had a second degree relative born with CL/P. There were only 7 cases (0.05%) of syndromic CL/P: Down's (4), Pierre Robin's (2), and Van der Woude Syndrome (1). A relationship was found between CL/P and the risk factors: taking anticonvulsants (without specifying the drug) (p = 0.025, OR = 10.73 C.I. 95%), taking retinoic acid (p-value = 0.049, OR = 4.75 C.I. 95%), not consuming folic acid (p-value = 0.00, OR = 28.23 C.I. 95%), and smoking cigarettes (p-value = 0.046, OR = 2.00 C.I. 95%). There was no relationship with maternal alcohol consumption or maternal diabetes (p-values = 0.652 and 0.210, respectively). SOM was present in 63.2% of patients with CL/P and were mostly isolated CP.

**Conclusion:**

CL/P was only slightly higher among males. The most common condition was CLP. There was higher incidence of CL/P among second-degree relatives than first degree. Down's, Pierre Robin's, and Van der Woude Syndromes may be associated with CL/P. Taking anticonvulsants, taking retinoic acid, not consuming folic acid, and smoking cigarettes all have a role in the incidence of CL/P. More than half of the sample had an associated SOM.

## 1 Introduction

Cleft lip and/or palate (CL/P) is one of the most common congenital defects, as it affects around 2 of every 1000 live births [1]. The Reported incidence of this condition varies between ethnic groups, with the highest prevalence seen in Asia, and the lowest in the Afro-Caribbean areas [2, 3, 4, 5]. As for morbidity and mortality, some research described increased mortality in patients with CL/P compared to those who are not [6, 7, 8]. CL/P has also shown to cause morbidity on the child and inflict both financial and societal burdens on the family [9]. CL/P can occur in an isolated form, or, less commonly, as a part of multiple chromosomal defects that results in different associated anomalies or a whole syndrome, which is then termed syndromic CL/P. Research has identified the genetic and environmental triggers for syndromic CL/P, but the aetiology of the non-syndromic form is still less identified [9]. The prevalence of CL/P also differs between genders. CL/P have shown to be more common in males than females in the ratio 2:1, while isolated CP is higher in females than males in the ratio 1:2. The anatomical distribution of the condition is asymmetrical, as bilateral clefts were found to be less common than unilateral clefts, and left-sided cleft lips occur more frequently than right-sided [10]. The occurrence of CL/P has been linked to several environmental and dietary risk factors. These risk factors include advanced maternal age, smoking, alcohol consumption, diabetes mellitus type I, deficiency of vitamins such as folic acid, and intrauterine irritation [11]. Moreover, maternal use of some drugs that are considered teratogenic such as valproate acid, anticonvulsants, retinoic acid derivates, thalidomide, and phenytoin also contribute to the incidence of CL/P [12, 13, 14, 15, 16, 17, 18, 19, 20]. Serous otitis media is a common finding in children with cleft palate. This is due to the disrupted insertion of the tensor veli palatini muscle on the soft palate. This muscle is responsible for opening the eustachian tube during swallowing, and therefore CP causes a functional obstruction in the tube and a subsequent negative pressure in the middle ear [21]. Our research aimed to study the gender and anatomical distribution of CL/P, as well as evaluate the risk factors, family history and association with serous otitis media.

## 2 Materials and methods

A case-control study was conducted on patients referred to two hospitals in Damascus, Syria: Damascus Hospital in the pediatric surgery, oral and maxillofacial surgery, and plastic surgery departments; and Al Zahrawi Hospital for obstetrics and gynaecology.

### 2.1 Participants

The case-control study sample included 133 patients who were present in Damascus Hospital, whether in clinics or wards, from 2016 until 2019, and in Al-Zahrawi Hospital in 2019.

### 2.2 Case-group sample

133 children born with cleft lip and/or cleft palate, with an ENT report of the diagnosis and classification, as well as a pediatric report screening for any associated syndromes, and had accessible data about the risk factors of CL/P screened for in our study.

### 2.3 Control-group sample

133 children born full-term in Al-Zahrawi Hospital for obstetrics and gynaecology without cleft lip and/or cleft palate as reported by Pediatrician, that had all the data about the risk factors of CL/P screened for in our study reported in their Gynaecology files.

### 2.4 Data acquisition

A form was prepared to fill in data about the patients' gender, origins, family history of cleft palate, consanguineous marriages, maternal risk factors (consumption of anticonvulsants, retinoic acid, alcohol, lack of folic acid consumption, smoking, and diabetes) and an ENT consultation for serous otitis media. As for the control group, the form included data about gestational age and maternal risk factors. The information was gathered by reading the patient's file and by referring to the resident doctors supervising the cases. The forms were then filled out and entered into the Excel data program and a comparison was made in terms of risk factors assumed for healthy newborns. The sample size is based on the total amount that accepted to participate in the case-group.

### 2.5 Statistical analysis

For statistical analysis, IBM SPSS Statistics v.25 software was used. The Goodness of Fit Test and Chi-Square were performed to determine the significance of the results, and multiple logistic regression was then carried to find out which of the independent variables studied most influence the dependent variable. Statistically significant results were reported for p-value < 0.05.

### 2.6 Ethical considerations

Written informed consent was taken from all participants. The ethical approval also took from the faculty of medicine Syrian Private University, in addition to the approval from Damascus hospital and Al-Zahrawi hospital.

## 3 Results

Table (1) The distribution of the sample according to gender showed that male participants were 51.9% while, females were 48.1%.

**Table 1:** Cleft lip and palate overview.

		N	%
Gender	Male	69	**51.9**
	Female	64	48.1
	**Total**	**133**	**100**
Cleft type	Isolated cleft lip	31	23.3
	Isolated cleft palate	34	25.6
	Cleft lip and palate	56	**42.1**
	Tessier	12	9
Cleft lip-type	Microform	7	7.1
	Incomplete	41	41.4
	Complete	51	**51.5**
	**Total**	**99**	**100**
Cleft lip classification	Left unilateral cleft	38	**38.4**
	right unilateral cleft	24	24.2
	central unilateral cleft	2	2
	Two-side	35	35.4
	**Total**	**99**	**100**
Type of cleft palate	Submucous Cleft	12	13.3
	Complete Cleft	36	40
	Incomplete Cleft	42	**46.6**
	**Total**	**90**	**100**

Regarding to the distribution of the study sample according to the type of cleft the most common type was cleft lip and palate by 42.1%. whereas, isolated cleft palate and isolated cleft lip were 25.6%, 23.3% respectively.

The distribution of the study sample according to the type of cleft lip the most common type was the complete with 51.5% of the cases, and the less was the microform with only 7.1% of total.

About the classification for the cleft lip, left unilateral cleft were the most viewed followed by thew tow side classification 38.4%, 35.4% respectively.

Cleft palate type distribution among the study sample showed that the incomplete cleft palate was the most common type 46.6%. then the complete cleft with 40%, and lastly the submucous cleft 13.3% of the total. (All the additional data were listed in the Table) (see Tables 2, 3, 4, 5, 6, 7, 8, 9, 10, 11).

**Table 2:** The distribution of cleft lip subtypes according to gender.

		Gender				Total	
		Male		Female			
		N	%	N	%		
Cleft lip classification	Left unilateral cleft	23	23.2%	15	15.2%	38	38.4%
	right unilateral cleft	13	13.1%	11	11.1%	24	24.2%
	central unilateral cleft	2	2.0%	0	0%	2	2%
	Two-side	19	19.2%	16	16.2%	35	35.4%
	Total	**57**	57.5%	**42**	42.5%	99	100%

**Table 3:** The distribution of cleft palate subtypes according to gender: the most common cleft palate classification in males was complete cleft 17.8%m while in females’ population the most common classification was incomplete cleft with 33.3% of total.

		Gender				Total	
		Male		Female			
		N	%	N	%		
the classification of cleft palate	Submucous Cleft	7	7.8%	5	5.5%	12	13.3%
	Complete Cleft	16	**17.8%**	20	22.2%	36	40%
	Incomplete Cleft	12	13.3%	30	**33.3%**	42	46.6%
	**Total**	**35**	**38.9%**	**55**	**61.1%**	**90**	**100**

**Table 4:** The distribution of the study sample according to the presence of consanguineous marriages between parents of patients.

		N	%
Consanguineous marriages	**No**	85	**63.9**
	**Yes**	48	36.1
	**Total**	**133**	**100**

**Table 5:** The distribution of the study sample according to the family history of cleft lip and palate and its degree.

		N	%
Family history	None	71	53.4
	First Degree	29	21.8
	Second Degree	33	24.8
	**Total**	**133**	**100**

**Table 6:** Some risk factors of cleft lip and palate commonly used during pregnancy.

		N	%
Risk factors	Take anticonvulsants	10	7.5
	Not taking folic acid	98	73.7
	Take retinoid acid	9	6.8
	Smoking	27	20.3
	Alcohol	3	2.3
	Diabetes	11	8.3

**Table 7:** Distribution of the syndromes that were associated with cleft lip and palate.

		N
Syndrome	Down	4
	PIERRE ROBIN	2
	Van der Woude	1
	Total	7

**Table 8:** The assumed risk factors for the control group of the same size as the cases sample (133).

		N	%
Risk factors	Take an anticonvulsant	1	0.75
	Not taking folic acid	12	6.8
	Take retinoid acid	2	1.5
	Smoking	15	11.3
	Alcohol	2	1.5
	Diabetes	6	4.5

**Table 9:** Analytical study of risk factors for cleft lip and palate using Chi-Square test. There is a statistically significant relationship between the fields which its p-value is less than (0.05). all the mentioned values are bold in the following table (taking anticonvulsant, not taking folic acid, cigarette smoking).

risk factor	Group	N	%	Chi-Square value	P-value
Take anticonvulsants	Cases group (n = 133)	10	**7.5**	7.681	**0.006**∗
	control group (n = 133)	1	0.75		
Take retinoid acid	Cases group (n = 133)	9	6.8	29.432	0.049∗
	control group (n = 133)	2	1.5		
Not taking folic acid	Cases group (n = 133)	98	**89.1**	67.236	**0.000**∗
	control group (n = 133)	12	10.9		
Cigarette smoking	Cases group (n = 133)	27	**20.3**	4.071	**0.044**∗
	control group (n = 133)	15	11.3		
Drinking alcohol	Cases group (n = 133)	3	2.3	0.204	0.652
	control group (n = 133)	2	1.5		
Diabetes	Cases group (n = 133)	11	8.3	1.571	0.210
	control group (n = 133)	6	4.5		

∗ There is statistical significance at (p-value≤0.05).

**Table 10:** Results of the logistic regression and the effect of all independent (studied) variables on the model that predicts cleft lip and palate incidence.

The accused risk factor		S.E.	p.value	OR	95% C.I. for OR	
					Lower	Upper
Cleft lip and palate injury versus no injury						
Anticonvulsants (Yes vs No)	2.373	1.056	0.025	10.732	1.354	85.068
Take retinoid acid (Yes vs No)	1.559	0.792	0.049∗	4.754	1.007	22.437
Not taking folic acid (Yes vs No)	3.341	0.361	0.000∗	28.233	13.913	57.294
Smoking (Yes vs No)	0.695	0.349	0.046∗	2.004	1.012	3.969
Alcohol (Yes vs No)	0.413	0.921	0.654	1.512	0.248	9.195
Diabetes (Yes vs No)	0.646	0.523	0.217	1.908	0.685	5.321

∗ There is statistical significance at (p-value≤0.05 (Unadjusted estimates of the odds ratio).

**Table 11:** The distribution of the study sample patients according to the presence of Serous otitis media: out of 56 participants with cleft lip and palate 36 of them had serous otitis media. And out of the total number (133 cases), 84 of them review with serous otitis media (63.2%).

		cleft type			Total cases of (SOM)
		Isolated cleft lip	Isolated cleft palate	Cleft lip and palate	
Serous otitis media	N	18/31	30/34	36/56	84/133
	%	58%	88.2%	64.2%	63.2%

## 4 Discussion

Cleft lip and/or palate (CL/P) are among the most common birth defects around the world, with a prevalence of 1.43 case for every 1000 live births [7]. In Syria, there is no exclusive database for patients with CL/P, and therefore we did not study the incidence rate in our country as we could not access all cases. Regional distribution of CL/P may vary between countries, as shown in several studies [22, 23, 24, 25]. In our sample, we found that most cases of condition are in the southern regions of Syria, at a rate of 76.7%.

When studying all cases with all types of clefts, we found that the condition was only slightly higher among males (51.9%) compared to females (48.1%) (Table 1). This distribution was similar to many similar studies^,^ such as one that showed a percentage of males of 53.85% [26], another of 55.1% [2], and a third had 52.9% males [27]. Some studies, on the other hand, showed no statistically significant difference in the occurrence of clefts between genders [23, 24, 28].

The most common condition in our sample was cleft lip and palate (CLP), 42.1%, followed by isolated cleft palate (CP), 25.6%, isolated cleft lip (CL), 23.3%, and only 9% were craniofacial cleft (Tessier). Most studies in the literature showed a similar distribution of the clefts [25, 28, 29], others also showed a dominance of the CLP, but the second most common cleft was CL rather than CP [27, 30]. Other fewer studies, on the other hand, had CP as the most common anomaly [26]. The distribution of the type of lip cleft in our study sample was as follows: the majority were complete cleft 51,5%, a little less incomplete cleft 41.1% and the minority were microform (7.1%). Regarding the location of the cleft lip, 35.4% of the cases were two-side cleft, 32.3% were right unilateral cleft, 28.3% were left unilateral cleft and 4% central unilateral cleft. Similarly, the majority of research results showed that unilateral cleft was more frequent than bilateral [2, 22, 25, 27, 29, 30]. One of the studies also showed a higher occurrence of right unilateral CL [27], while another found dominance in the left side [22].

In our sample, the patients with cleft palate were mostly complete cleft (46.6%), while the incomplete cleft constituted 40% of the rest were submucosal (13.4%). A study in Estonia also showed that the most common cleft type was incomplete cleft palate [29].

We grouped the cases according to the types of clefts and studied the gender distribution, and the results were as follows: Isolated cleft lip (CL) was only slightly higher in males (51.6%) than females (48.4%), while combined cleft lip and palate (CLP) had a much higher incidence of 62.5% in males, compared to females (35.7%). On the other hand, both Tessier condition and isolated cleft palate (CP) were more prevalent among females (58.3% and 64.7% respectively), except the submucosal subtype of CP that was higher among the males. Other studies also found a higher incidence of CP among females [26, 27, 28], and a higher incidence of CLP among males [25, 27, 28], one of which had a male dominance for CL [25]. Contrary to our study, one paper showed that CL was much higher among females (70%) [26], while another found no significant difference between the incidence of CL between genders [24].

We found that 36.1 % of the parents of the cases in our study were relatives. A study in Iran showed a very similar percentage as 33.7% of the cleft patients in their sample were born from consanguineous marriages [2].

We studied the family history of CL/P for all patients along with the degree of relation to a family member with CL/P, and results showed that 21.8% of cases had a first-degree relative and 24.8% had a second-degree relative with CL/P, while the remaining 53.4% had no family history of CL/P. Papers that studied the link between CL/P and family history found a significant relationship [2, 22, 25, 30], one of which also found a positive family history in 27.6%, and especially second degree relatives [22].

Research states that patients with clefts may have associated anomalies with a frequency ranging from 44%-66% and that isolated cleft palate is the defect with the highest rate of associated anomalies [31]. CL/P may also be a part of a whole syndrome and is then termed syndromic CL/P. The two most common syndromes associated if orofacial clefts are Van der Woude and Pierre Robin sequence [31, 32]. Van der Woude's defects are usually bilateral isolated CL [32], while Pierre Robin's are mostly CP [31].

In our paper, we studied the associated anomalies as a whole syndrome or sequence, and our sample included only 7 cases (0.05%) of syndromic CL/P distributed as follows: four cases of Down's Syndrome, two of which were complete isolated CP and two CLP. In the cases with combined CLP, one of the cases had a central complete CL and the second had a complete right CL, while both CP cases were incomplete CP; two cases of Pierre Robin syndrome, both of which were complete isolated CP; and one case of Van der Woude Syndrome as bilateral complete isolated CL.

In order to study the risk factors for CL/P, such as maternal consumption of anticonvulsants, retinoid acid, and alcohol, not taking folic acid, and smoking, the statistical differences between the two groups (case group, control group) were studied by applying the Goodness of Fit Test and calculate its (Chi-Square) statistic to equal proportions. multiple logistic regression of the CL/P incidence variable on the independent variables was then carried to find out which of the independent variables studied most influence the dependent variable (cleft lip and palate).

Results showed no statistically significant relationship between both maternal alcohol consumption and maternal diabetes and cleft lip and palate, with p-values = 0.652 and 0.210, respectively. On the contrary, some studies did find a relationship between these two factors and the incidence of CL/P [22, 25, 29].

On the other hand, a statistically significant relationship was found between cleft lip and palate and the risk factors: taking anticonvulsants (without specifying the drug) (p = 0.025), taking retinoic acid (p-value = 0.049), not consuming folic acid (p-value = 0.00), and smoking cigarettes (p-value = 0.046). According to the multiple logistic regression model in our study, the absence of folic acid consumption increases the incidence of CL/P by 28 times (OR = 28.23 C.I. 95%), while consumption of anticonvulsants increases the likelihood by 10 times (OR = 10.73 C.I. 95%), and consumption of retinoic acid increases it by 4 times (OR = 4.75 C.I. 95%). Our model also showed that infants born from a smoking mother are only two times more likely to have CL/P (OR = 2.00 C.I. 95%) that those who are born from a non-smoking mother. Similar to our results, several studies in the literature also found a significant association between CL/P and the lack of folic acid intake [2, 22, 27] and smoking [30]. Although studies did prove the role of folic acid in DNA and protein synthesis and the protective effect against CL and CLP, the specific mechanism through with the lack of B9 predisposes to both facial defects, neural tube defects and other congenital malformations remains under research [33]. Research on retinoic acid demonstrated that despite its important roles during palate development, excess retinoic acid is teratogenic and causes both cleft palate and other malformations in fetuses of both animals and humans [34, 35]. As for maternal use of anticonvulsants, other papers also found a significant relationship to CL/P [36, 37], one of which deduced that fetuses from mother taking anticonvulsants are ten times more likely to have CL/P [37], just like our results.

In our sample, 63.2% of patients with cleft palate were diagnosed with SOM, and the cleft associated with the highest incidence of SOM has isolated CP, 88.2%, i.e., 88.2% of patients with isolated CP had SOM. Similarly, a study in Belgium also detected the presence of otitis media in about 62% of children aged below 6 years old [38].

### 4.1 Limitations and obstacles

The Covide 19 pandemic and its effect with the lockdown and lifestyle. The difficulty to reach the information and the participants data due to the Syrian crisis. Some of them leave the country.

## 5 Conclusion

The most common condition in our sample was CLP, followed by isolated CP, isolated cleft lip and Tessier. CL/P was only slightly higher among males and much higher among second-degree relatives. Cases of syndromic CL/P were distributed between Down's, Pierre Robin's and Van der Woude Syndrome. Taking anticonvulsants, taking retinoic acid, not consuming folic acid, and smoking cigarettes were related to the incidence of CL/P. The highest contributing factor was the lack of folic acid consumption, which, hence, should be added to the diet of all pregnant women. Retinoic acid and smoking should be avoided. Women on anticonvulsants should consult their physician about switching the drug during pregnancy to a safer form, such a Lamitrigine or Levetiracetam. More than half of the sample had an associated SOM, which highlights the importance of its screening in all patients with CL/P.

## Declarations

### Author contribution statement

Louei Darjazini Nahas and Omar Alzamel: Conceived and designed the experiments.

Mammdouh Yassin Dali and Rama Alsawah: Performed the experiments.

Rafi Sulman and Mohamad Alzamel:Analyzed and interpreted the data.

Ahmad Hamsho: Contributed reagents, materials, analysis tools or data.

Abdullah Mohamad Omar: Contributed reagents, materials, analysis tools or data; Wrote the paper.

### Funding statement

This research did not receive any specific grant from funding agencies in the public, commercial, or not-for-profit sectors.

### Data availability statement

Data included in article/supplementary material/referenced in article.

### Declaration of interests statement

The authors declare no conflict of interest.

### Additional information

No additional information is available for this paper.
